# B-cell leukemia transdifferentiation to macrophage involves reconfiguration of DNA methylation for long-range regulation

**DOI:** 10.1038/s41375-019-0643-1

**Published:** 2019-11-12

**Authors:** Alberto Bueno-Costa, David Piñeyro, Marta Soler, Biola M. Javierre, Helena Raurell-Vila, Marc Subirana-Granés, Lorenzo Pasquali, Jose A. Martinez-Climent, Manel Esteller

**Affiliations:** 1 Josep Carreras Leukaemia Research Institute (IJC), Badalona, Barcelona, Catalonia Spain; 2 Program of Predictive and Personalized Medicine of Cancer (PMPPC), Endocrine Regulatory Genomics Laboratory, Department of Endocrinology and Nutrition, Germans Trias i Pujol University Hospital and Research Institute (IGTP), Badalona, Catalonia Spain; 3 CIBER de Diabetes y Enfermedades Metabólicas Asociadas (CIBERDEM), Barcelona, Spain; 4 Division of Hematological Oncology, Center for Applied Medical Research (CIMA), University of Navarra, Pamplona, Spain; 5 Centro de Investigacion Biomedica en Red Cancer (CIBERONC), 28029 Madrid, Spain; 6 Institucio Catalana de Recerca i Estudis Avançats (ICREA), Barcelona, Catalonia Spain; 7 Physiological Sciences Department, School of Medicine and Health Sciences, University of Barcelona (UB), Barcelona, Catalonia Spain

**Keywords:** Haematopoietic stem cells, Acute lymphocytic leukaemia

## To the Editor:

Hematopoiesis is a highly regulated process that, starting from hematopoietic stem cells (HSCs) with self-renewal capacity in the adult human bone marrow, is able to generate all different types of mature blood cells. The classical view of hematopoiesis defines binary branching points from these HSCs that segregate lineages and direct differentiation to terminally differentiated functional cell types [1]. However, the described hierarchical model can be complemented with the emerging data that suggest the existence of hematopoietic stem and progenitor cells with a continuum of transitory differentiation stages, including cells with early lineage priming that generate distinct blood cell types according to the physiological or pathological environment [2]. In this context, there are increasing data of hematopoietic plasticity and cell lineage conversion, particularly in leukemogenesis. Examples of transdifferentiation include B-cell lymphomas that can transform to histiocytic/dendritic cell sarcoma, erythroid/megakaryocytic lineages changing to granulomonocytic-like lineage upon use of a histone demethylase LSD1 inhibitor or B-ALL (acute lymphoblastic leukemia) patients that evaded CD19-directed antibody therapy (blinatumomab) by undergoing myeloid-lineage switch. Related to the latter scenario, lineage switching has also been reported as a cause of antigen loss in chimeric antigen receptor T-cell therapies, where B-ALL patients transdifferentiate in their relapse as acute myeloblastic leukemia in response to the initial CD19-directed immunotherapy [3]. Due to the central role of epigenetics, particularly DNA methylation, in the successful generation of differentiated blood cell types and its plasticity during lineage specification [4], we wondered about its function in hematopoietic transdifferentiation, a largely unexplored field.

**Fig. 1:**
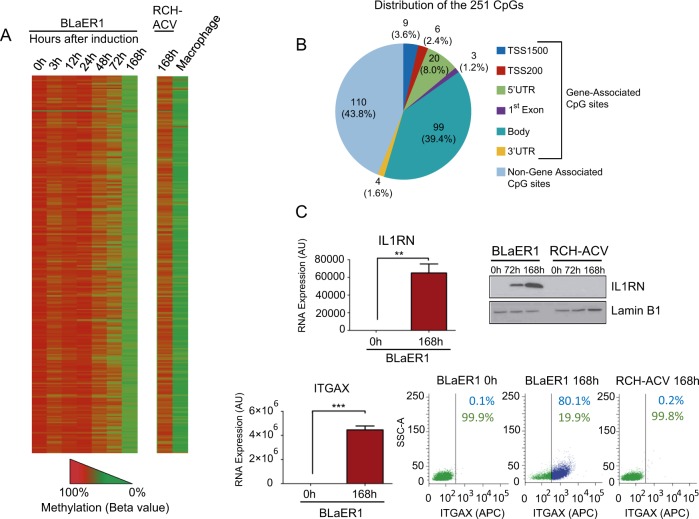
DNA methylation analysis at different timepoints of B-ALL-to-Macrophage transdifferentiation. **a** Heatmap showing the methylation state of the 251 significant hyper/hypomethylated CpGs during 7 days of transdifferentiation. RCH-ACV (treated 7 days with 17β-estradiol, IL-3, and M-CSF) and macrophage are taken as negative and positive controls, respectively. **b** Pie chart showing the genomic distribution of the 251 significant CpGs. **c** qRT-PCR (***p < 0.001, *T*-Test) western blot and flow cytometry analysis at different timepoints of transdifferentiation of IL1RN and ITGAX, whose associated promoter CpGs are significantly demethylated during transdifferentiation

Our studied model of transdifferentiation is a well-defined experimental system that converts B cells into macrophages. Following initial work that demonstrated that normal murine B-cell precursors as well as mature antibody-producing B cells can be induced by C/EBPα to transdifferentiate into functional macrophages [5], a murine cellular model was established of pre-B cells containing a fusion of C/EBPα with the estrogen receptor hormone binding domain (C/EBPαER) that converts them to macrophage-like cells upon 17β-estradiol exposure [6]. We have recently translated this model to human B-lymphoma and leukemia cell lines that can be induced by C/EBPα to transdifferentiate into functional macrophages [7]. Importantly, primary human BCR-ABL1(+) B-ALL cells could also be induced to reprogram into macrophage-like cells by transient expression of C/EBPα [8]. To explore the changes that the DNA methylome undergoes upon transdifferentiation, we have herein applied this experimental system. Thus, we have analyzed the human precursor B-ALL cell line RCV-ACH transfected with the transgene C/EBPαER, thereafter termed BLaER1, upon 17β-estradiol-mediated transdifferentiation at seven timepoints (0, 3, 12, 24, 48, 72, and 168 h) using a comprehensive DNA methylation microarray that interrogates more than 850,000 CpG sites (Supplementary Fig. 1a and Supplementary Methods). DNA methylation data are available on the GEO repository under accession number GSE132845. We have observed a significant change in the methylation status of 251 CpG sites during the transdifferentiation process (*p*-value < 0.05 and CpG *B*-value change ≥0.66) (Supplementary Table 1 and Supplementary Methods). Most strikingly, all except one (250 of 251, 99.6%) were hypomethylation changes (Fig. 1a and Supplementary Fig. 1a). In this regard, these hypomethylation events occurred in the context of downregulation of the DNA methyltransferases DNMT1 and DNMT3B, but not DNMT3A, in our transdifferentiation model (Supplementary Fig. 2). The DNA methylation pattern of the endpoint of transdifferentiation (BlaER1 at 168 h) for these sites mimicked the CpG methylation status of naive macrophages (Fig. 1a and Supplementary Table 1). According to genomic distribution of the identified CpG sites, 141 CpGs (56.2%) had an associated gene, whereas 110 CpGs (43.8%) were in regions of the genome without any annotated gene (Fig. 1b).

Due to our interest in epigenetic modifications that can actively contribute to the transdifferentiation phenomenon and the classical view of the impact of DNA methylation on transcription, we first studied the 141 CpG sites that are annotated as having an associated gene in relation with the available expression levels of the corresponding gene [9]. We identified 41 CpG sites (29.1%), corresponding to 39 genes (Supplementary Table 2), for which the methylation status significantly correlated with the expression of the associated gene: in most cases the CpG hypomethylation event was linked to gene expression (32 of 39, 82%), whereas only in a minority of cases was demethylation associated with gene repression (7 of 39, 18%) (Supplementary Table 2). Using data mining (Supplementary Methods), we observed that these 41 CpG sites were in binding sites for 80 transcription factors (Supplementary Fig. 3a). Gene ontology analysis using a hypergeometric test to find biological processes overrepresented in our set of transcription factors (Supplementary Methods) demonstrated, in addition to global regulatory networks, an enrichment in the “immune system development,” “cell fate commitment,” and “leukocyte differentiation” categories (Supplementary Fig. 3b), including homeobox proteins related to leukemia biology (such as MEIS1, HOXA2, and HOXC11) and lymphoid and myeloid-differentiation-associated programs (such as FOXC1, GATA2, and SPI1). We proceeded further to validate two candidate genes detected as undergoing CpG demethylation-associated reactivation for our multiomic approaches in the transdifferentiation model: the interleukin-1 receptor antagonist (IL1RN) and integrin alpha X (ITGAX), both genes that are almost exclusively expressed in macrophages [10, 11]. The methylation changes of the identified CpG sites associated with IL1RN and ITGAX were further confirmed by bisulfite genomic sequencing of multiple clones and bisulfite pyrosequencing (Supplementary Fig. 4 and Supplementary Methods). Quantitative real-time PCR (Supplementary Methods) demonstrated lack of expression for both transcripts in the initial B-ALL cells in the absence of 17b-estradiol treatment and a strong gain of expression at the end of the transdifferentiation process in the macrophage-like cells (Fig. 1c). The assessment of protein levels for IL1RN (western blot) and ITGAX (cell cytometry) (Supplementary Methods) obtained the same expression patterns (Fig. 1c). The use of the demethylating agent 5-aza-2′-deoxycytidine in BLaER1 cells induced IL1RN and ITGAX expression (Supplementary Fig. 5), supporting the role of CpG methylation in gene silencing. Most importantly, 17β-estradiol treatment of the original naive pre-B-ALL leukemia cells RCH-ACV, which were not transfected with the transgene C/EBPαER and thus do no transdifferentiate, maintained the gene silencing of IL1RN and ITGAX (Fig. 1c). These data support the role of a wave of DNA demethylation that, acting locally in the CpG-associated genes, confers macrophage identity to the original pre-B-ALL cells.

**Fig. 2:**
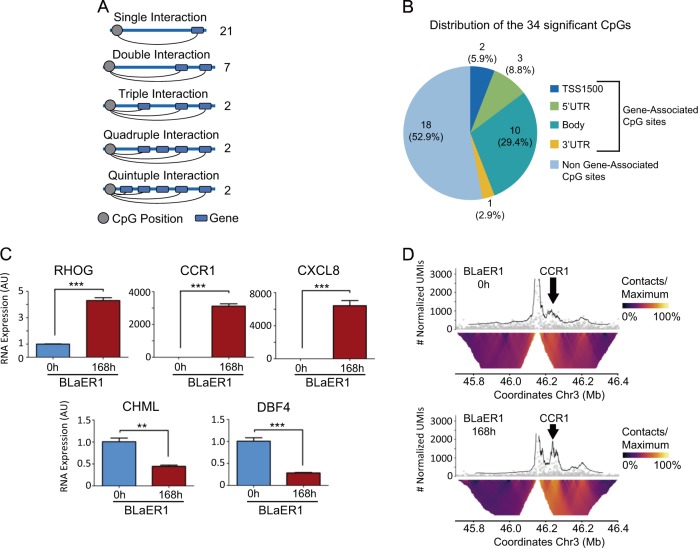
Analysis of the loop formation between differentially methylated distant regions and gene promoters. **a** Illustrative representation of CpG interaction with promoter regions that significantly modulated expression after transdifferentiation. **b** Distribution of the 34 significant CpGs on the human genome. **c** RT-qPCR (*p < 0.05, ***p < 0.001, *T*-Test) of three enhancer (top) and two candidate silencer-controlled genes (below). **d** View of the 3D chromatin contacts, obtained by UMI-4C, for a bait located at the CpG-containing candidate enhancer sequence for the CCR1 promoter in the BlaER1 transdifferentiation model. The top graph of the panel shows the smoothed trend line of the contact profiles of the bait at time 0 (top) and at the end of BlaER1 transdifferentiation (168 h) (below). A bottom heatmap (domainogram) reports the differential mean contacts per a series of restriction enzyme fragments. The UMI-4C analysis shows that upon BlaER1 transdifferentiation the hypomethylated CpG-containing sequence engages a strong distal chromatin contact (black arrow) with the promoter of CCR1. Of note the chromatin contact intensity for the same region was very low for the hypermethylated regulatory sequence at time 0 h of transdifferentiation (Chi-square adjusted *p* < 0.0001)

Strikingly, our analysis workflow showed, as described above (Fig. 1b), that among the differentially methylated CpGs in our transdifferentiation model, 110 CpGs (43.8%) were located in genome contexts without any genes in their vicinity. Thus, in these cases, it is possible that a candidate regulatory event of those CpGs occurs in the 3D organization of the human genome. In this regard, regulation at long-range distance can occur due to the formation of “loops” in the DNA that, for example, collocate enhancer or silencer sequences and minimal promoters. DNA methylation patterns at enhancers are relevant to the determination of cell identity [12] and their aberrant CpG methylation profile has been observed in human cancer [13], including hematological malignancies. Thus, we investigated how many of these nongene associated CpGs were located in distant regulatory regions, taking advantage of our available Promoter Capture Hi-C (PCHi-C) data in macrophages [14]. We also included in our analyses the 141 CpG sites with associated genes due to the possibility that, in addition to regulating genes in lineal proximity, these CpG sites could also act as long-range interactor sequences. We found that 72 of the overall 251 CpGs (28.7%) were located in candidate distant regulatory regions defined by PCHi-C in macrophages (Supplementary Fig. 1b and Supplementary Table 3) [14]. To address the functionality of the observed DNA methylation changes, we then interrogated whether the methylation status of these 72 CpG sites had any impact on the expression of the genes whose promoters are targeted by these long-range regulatory sequences [14]. We found that the methylation status of 34 of the 72 CpG sites (47.2%) correlated with the expression of 52 genes that interacted with these regions (Supplementary Fig. 1b and Supplementary Table 4). Importantly, all except one (33 of 34, 97%) were CpG hypomethylation changes (Supplementary Table 4). Most of the 34 PCHi-C-derived CpG dinucleotides represented unique interactions between one methylation site and one gene (21 of 34, 62%) (Fig. 2a), followed by dual interactions (one CpG interacting with two genes, 7 of 34, 21%), but more complex interactions were also observed (Fig. 2a). Interestingly, although many of these CpG sites were in nonassociated gene regions (18 of 34, 53%), we also frequently observed CpGs within associated genes (16 of 34, 47%) (Fig. 2b). It is important to highlight that the CpGs in the latter subset were mainly located in nonpromoter regions of the gene (11 of 16, 69%) (Supplementary Table 4). Using data mining (Supplementary Methods), we observed that four of the PCHi-C-derived CpG dinucleotides contained a binding motif for the CCCTC-binding factor (CTCF), the most widely recognized protein controlling three-dimensional structures of DNA. Related to the 52 identified target genes, for 31 genes (60%) the CpG hypomethylation event acted as an enhancer event associated with the expression of the corresponding gene, whereas for 21 genes (40%) the demethylation event was associated with gene inactivation and can be classified as a candidate silencer sequence in our pre-B-ALL to macrophage transdifferentiation model (Supplementary Fig. 1b and Supplementary Table 4).

We experimentally validated the available expression microarray data [7] using quantitative real-time PCR for five candidate targets: three genes with CpG hypomethylation associated expression upon transdifferentiation (ras homolog family member G, RHOG; C-X-C motif chemokine ligand 8, CXCL8; and C-C motif chemokine receptor 1, CCR1) and two genes with CpG demethylation-associated expression reduction upon cell conversion (CHM like Rab escort protein, CHML; and DBF4 zinc finger, DBF4) (Fig. 2c). The methylation changes of the distant CpG sites associated with the target genes was further confirmed by bisulfite genomic sequencing of multiple clones and bisulfite pyrosequencing (Supplementary Fig. 6 and Supplementary Methods). The use of the demethylating agent 5-aza-2′-deoxycytidine in BLaER1 cells induced RHOG, CXCL8, and CCR1 expression (Supplementary Fig. 5), further supporting a role of the identified CpGs in the candidate enhancer sequences. Finally, using targeted chromosome capture with unique molecular identifiers (UMI-4C), a recently developed method to quantitatively measure 3D interaction intensities between distant regulatory regions and their corresponding promoters [15], we decided to confirm the loop dynamics of the singled-out targets. One technical limitation of UMI-4C is that it provides reliable contact quantifications only between DNA sequences located within a ~0.5 Kb and ~1 Mb interval [15]: among the five studied genes, only the CCR1 promoter and its putative PCHi-C obtained CpG-containing regulatory region fulfilled these parameters. UMI-4C data are available at the SRA repository under accession number PRJNA548887. Most importantly, we found that the CpG demethylation event occurring during transdifferentiation for the PCHi-C derived candidate long-distance regulatory sequence of CCR1 was not only associated with the expression of the gene (Fig. 2c), but also with the formation of a new loop between the CpG-containing distant regulatory region and the CCR1 proximal promoter (Chi-square test *p* < 0.0001) (Fig. 2d), supporting a role as an enhancer. These data highlight the relevance of DNA methylation events at distant regulatory regions to confer cell identity for both B-ALL and macrophage cells.

In conclusion, we report that transdifferentiation events in the context of hematopoietic lineage plasticity, such as the pre-B-ALL lineage conversion to macrophage studied herein, involve DNA methylation shifts that not only affect CpG sites in lineal proximity to genes, but also incur epigenetic changes in long-range interactor sequences derived from the 3D genome architecture of the living cell. These results may help to improve our knowledge of the critical determinant for cell type specification and to understand what goes awry in hematological malignancies that, in response to pharmacological or cellular therapies, undergo lineage switching to develop resistance to the applied treatment.

## Supplementary information

Supplementary Methods

Supplementary Figure 1

Supplementary Figure 2

Supplementary Figure 3

Supplementary Figure 4

Supplementary Figure 5

Supplementary Figure 6

Supplemental Table 1

Supplemental Table 2

Supplemental Table 3

Supplemental Table 4
