# Supplementary material for: B-cell leukemia transdifferentiation to macrophage involves reconfiguration of DNA methylation for long-range regulation
Source: Leukemia. 2019 Nov 12;34(4):1158–62. doi: 10.1038/s41375-019-0643-1 (PMC7214273; doi:10.1038/s41375-019-0643-1)
Supplement: Supplementary file 1 — Supplementary Methods [file 41375_2019_643_MOESM1_ESM.docx]

**Supplementary Methods**

**Cell lines and macrophages**

BLaER1 and RCH-ACV cell lines were provided by Thomas Graf group.^1^ These cells were cultured at 5% CO_2_, 37°C with RPMI-1640 GlutaMAX (Gibco, Ref: 61870-044), 10% FBS (Gibco, Ref: 16000-044), 1X Penicillin/Streptomycin (BioWest, Ref: L0022-100). All cell lines were authenticated by short tandem repeat profiling (LGS Standards SLU) and tested for the absence of mycoplasma.

Macrophages were obtained from Macrophage colony-stimulating factor (MCSF) induced differentiation of monocytes as previously described.^2,3^ Briefly, an initial buffy coat with peripheral blood of an anonymous donor was obtained through the Catalan Blood and Tissue Bank (CBTB). The CBTB follows the principles of the Declaration of Helsinki (World Medical Association, WMA). Before providing the first blood sample, the donor received detailed oral and written information and signed a consent form at the CBTB. Pure monocytes were isolated from PBMCs using positive selection with MACS CD14 beads (Miltenyi Biotec, Bergisch Gladbach, Germany, Ref: 130-050-201). 5 x 10^6^ monocytes were then plated in 10 mL of serum-free RPMI-1640 GlutaMAX medium and incubated at 5% CO_2_, 37°C. After 2 hours of incubation, medium was replaced with 10 mL RPMI-1640 GlutaMAX, 10% FBS, 1X Penicillin/Streptomycin and 25 ng/mL of M-CSF (Peprotech, 300-25-50UG). Cells were cultured at 5% CO_2_, 37°C and harvested after 3 days of culture.

**Transdifferentiation of BLaER1 cells**

In order to induce the B-Cell-to-Macrophage transdifferentiation, 5 x 10^6^ BLaER1 cells were cultured in 10 mL RPMI-1640 GlutaMAX medium supplemented with 10% FBS, 1X Penicillin/Streptomycin, 100 nM of 17β-estradiol (Sigma, Ref: E8875), 10 ng/mL of IL3 (Peprotech, Ref: 200-03-50UG) and 10 ng/mL of M-CSF. BLaER1 cells were harvested at seven different time-points of transdifferentiation (0, 3, 12, 24, 48, 72, and 168 hours) and pellets were frozen at -80°C.

DNA from each time-point was purified as previously described.^4^ Briefly, cell pellets were lysed with 600 µL of lysis buffer (10 mM Tris-HCl, pH=7.4; 10 mM EDTA; 200 mM NaCl), 75 µL of 10% SDS and 15 µL of Proteinase K (20 mg/mL), incubated for 3 hours at 55 ºC. Proteinase K was inactivated by simple incubation at 75 ºC, 15 minutes. 2 µL of RNase A 10 mg/mL were added to each sample and incubated for 1 hour at 37 ºC. 300 µL of NaCl 5M were added to the samples and then were centrifuged for 5 minutes at 14000 g (25 ºC), collecting the supernatant. 0.1 volumes of AcNH_4_ 10 mM, 1 µL of GlycoBlue 15 mg/mL (Ambion, Ref: AM9515) and 1 volume of isopropanol 100% were added and centrifuged for 15 minutes at 4 ºC (maximum speed). The supernatant was discarded, the pellet was resuspended with 500 µL of Ethanol 70% and centrifuged 15 minutes at 4 ºC (maximum speed). The pellet was air-dried, resuspended in DNase-free water for 10 minutes at 55 ºC and stored at -20 ºC.

**DNA Methylation analysis**

The DNA methylation array used was the MethylationEPIC BeadChip 850K microarray.^5^ Genomic location of the CpGs under study are depicted in the table below. The term “Beta-value”, as included in the text, refers to the estimate of methylation level using the ratio of intensities between methylated and unmethylated alleles in the Illumina DNA methylation microarray. It does not refer to the statistical term. B-values for each CpG are between 0 and 1 with 0 being unmethylated and 1 fully methylated. Validation of the methylation state of promoters and distant regulatory regions was determined by bisulfite genomic sequencing (BSP), using EZ DNA Methylation Gold kit (Zymo Research, Orange, CA, USA, Ref: D5006) for DNA conversion and specific primers to amplify the regions of interest (see table below). Amplicons were cloned into the pGEM-T Easy Vector System I (Promega, Ref: A1360). Competent *E. coli* (DH5α strain) were transformed in LB-agar plates treated with ampicillin, X-Gal and IPTG. A minimum of six clones were selected to calculate the methylation frequency. Plasmid purification for each clone was performed using the NucleoSpin 96 plasmid kit (Macherey-Nagel, Ref: 740625.24). Amplicon sequencing was performed using the 3730 DNA analyzer (Applied Biosystems, Ref: 3730S) and methylation state for each clone was represented using BSmapR software. DNA methylation was also studied by pyrosequencing using the PyroMark Q48 Advanced Reagents kit (PyroMark Q48 Autoprep Pyrosequencer, Qiagen, Ref: 974002). T-Test for unpaired samples was used to perform statistical analysis. Normality and homogeneity in variance is assumed for Pyrosequencing experiments with biological triplicates. Primers are listed in the table below.

Illumina Infinium HumanMethylation EPIC raw data was loaded into R statistical language,^6^ to perform all the analysis. QC and pre-processing steps were performed using minfi package.^7,8^ Briefly, raw data was normalized using ssNoob algorithm. Then, probes with low detection p-value (< 0.01), probes with a known SNP (Single Nucleotide Polymorphism) at the CpG site and known cross-reactive probes,^9^ were removed. For the resulting CpGs, Beta- and M-values were calculated using minfi functions. Beta-value for an i^th^ interrogated CpG site is defined as:

$${Beta}_{i}=\frac{max\left( {methy}_{i},0 \right)}{max\left( {unmethy}_{i},0 \right)+max\left( {methy}_{i},0 \right)+\alpha}$$

where *methy_i_* and *unmethy_i_* are the intensities measured by the i^th^ methylated and unmethylated probes, respectively. Illumina recommends using a regularization parameter (α = 100) to avoid dividing by small numbers. However, in practice this is not necessary as most of the probes have intensities (*methy_i_* + *unmethy_i_*) larger than 1000.^10^ Therefore, this regularization parameter is not used by default (α = 0). The name “Beta-values” was chosen because they should follow approximately a Beta distribution, assuming that probe intensities are normally distributed.

M-value for an i^th^ interrogated CpG site is defined as:

$$M_{i}={log}_{2}\left( \frac{max\left( {methy}_{i},0 \right)}{max\left( {unmethy}_{i},0 \right)} \right)$$

The relationship between Beta- and M-values can be derived by substitution as follows:

$${Beta}_{i}=\frac{2^{M_{i}}}{2^{M_{i}}+1};M_{i}={log}_{2}\left( \frac{{Beta}_{i}}{1-{Beta}_{i}} \right)$$

It is known that Beta-values suffer from significant heteroscedasticity at both sides of their distribution. This problem is effectively solved after transforming Beta-values to M-values,^10^ making them more appropriated for subsequent statistical analysis, as the linear models explained below.

**DNA methylation changes upon differentiation treatment.**

M-values were used to fit linear regression models in order to find the more “methylation ~ treatment duration” correlated CpGs. Using R statistical language, the following linear model was implemented:

$$M_{pred}=\beta_{0}+\beta_{1}X+\varepsilon$$

where *M_pred_* is the predicted methylation M-value (response or dependent variable), *X* is the treatment time in hours (predictor or explanatory variable), *β_1_* is the estimated coefficient for the time variable, *β_0_* is the intercept and ε is the error term. In order to find the CpGs in which its methylation is more strongly associated to treatment duration, we filter out CpGs based on the following criteria:

1) The t-test p-value for the *β_1_* coefficient estimate should be below 0.05. This test provides a good notion of how far this coefficient is from 0 and therefore if the treatment is significantly affecting the methylation.

2) As a goodness of fit indication, the R-squared (coefficient of determination) was also required to be above 0.5. This statistic represents the proportion of the variability explained by the model.

3) To make sure that the magnitude of the methylation changes is biologically significant and far above the expected measuring error, a difference of 0.66 in Beta-value between the beginning and the end of the treatment was also required.

**DNA methylation ~ gene expression correlation.**

In order to investigate whether methylation changes found affected gene expression, we took advantage of previous gene expression data from the same model (Gene Expression Omnibus accession: GSE44700).^1^ Thus, a methodological limitation of the study is that DNA methylation and microarray expression were not evaluated in the same biological samples. Gene expression microarray raw data were processed using agilp package.^11^ From the 251 selected CpGs, 141 had an associated gene, i.e. were located in the gene or nearby the TSS (Transcription Start Site). Using this 141 CpGs subset and the aforementioned gene expression data, we then fit linear regression models to find significant “methylation ~ gene expression” correlations along the differentiation treatment.

Using R statistical language, a linear model similar to the previous one was implemented:

$$M_{pred}=\beta_{0}+\beta_{1}X+\varepsilon$$

where *M_pred_* is the predicted methylation M-value (response or dependent variable), *X* is now the log_2_ gene expression value (predictor or explanatory variable), *β_1_* is the estimated coefficient for the gene expression variable, *β_0_* is the intercept and ε is the error term. In order to find the CpGs in which its methylation is more strongly associated to expression, we filter out CpGs based on a similar criterion as previously:

1) The t-test p-value for the *β_1_* coefficient estimate should be below 0.05. This test provides a good notion of how far this coefficient is from 0 and therefore if the treatment is significantly affecting the methylation.

2) As a goodness of fit indication, the R-squared (coefficient of determination) was also required to be above 0.5. This statistic represents the proportion of the variability explained by the model.

**DNA methylation in distant regulatory regions.**

From the 251 CpGs found responding to the differentiation treatment and, taking advantage of the available PC-HiC data from a relevant macrophage 0 model, we investigated how many of those CpGs were located in enhancer/silencing active regions. We first selected 72 out of 251 CpGs located in putative distant regulatory regions found in the PC-HiC experiment. For this 72 CpGs subset, we investigated the correlation between methylation and expression of the distant genes found in contact by PC-HiC data, by fitting linear regression models using the same formulation as previously described (see the previous section: “DNA methylation ~ gene expression correlation” from supplementary methods). In this case, the *X* parameter (the log_2_ gene expression value) corresponds to distant genes found in contact by PC-HiC.

As a result, a total of 34 CpGs for which methylation and gene expression values were significantly correlated throughout the differentiation treatment. These 34 CpGs were located in 34 different distant regulatory regions and, as some of them were found in contact with more than a single promoter region, we found a total of 59 enhancer/silencing – promoter significant interactions: 38 putative enhancers, as methylation anti-correlated with gene expression; and 21 putative silencers, as methylation directly correlated with gene expression.

**Validation of the Gene Expression changes**

qRT-PCR, PCR, Western Blot and Flow Cytometry analysis were performed to confirm mRNA and protein expression changes of some of the most significant genes. For qRT-PCR, biological triplicates were harvested at different timepoints (0 hours vs 168 hours). RNA extraction was carried out using the RNAeasy mini kit (Qiagen) according to the manufacture guidelines. Retrotranscription of 2 µg of total RNA was performed using RevertAid RT kit (Thermo Scientific, Ref: 00719361). qRT-PCR was performed using the QuantStudio 5 system (Applied Biosystems, Ref: A28140) with the conditions listed in the table below. PGK was used as housekeeping gene as previously described.^1^ T-Test for unpaired samples was used to perform statistical analysis (T-Test, *p<0.05). Normality and homogeneity in variance is assumed for qRT-PCR experiments with biological triplicates. Primer sequences are listed in the table below.

Western blot was performed as previously described.^12^ Briefly, total protein extract was obtained from frozen cell pellets using RIPA 1X lysis buffer (PBS 1X, deoxycholate 0.5%, NP-40 1%, SDS 0.5% and 1 pill of protease/phosphatase inhibitor from Roche, Ref: 04 693 132 001), sonicated for 5 seconds and denatured for 5 minutes at 95 °C. Protein concentration was determined using the BCA assay (Pierce BCA Protein Assay Kit, Thermo Scientific, Ref: 23225). 40 µg of each protein sample was separated on 12-15% SDS-polyacrilamide gels by SDS-electrophoresis and transferred onto a 0.2 µm-porus nitrocellulose membrane (Amersham Protran 0.2 µm NC, GE Healthcare, Ref: 10600001) by wet electroblotting (Mini Trans-Blot Cell, Bio-Rad, Ref: 1703930) for 1 hour at 100 V. The membrane was blocked with 5% non-fat dry milk in PBS Tween-20 0.1% for 1 hour at room temperature. Primary antibodies were used overnight at 4 °C (dilution conditions are listed in the table below). Membranes were washed thrice for 7 minutes in PBS Tween-20 0.1% and incubated with secondary antibodies conjugated to horseradish peroxidase for 1 hour at room temperature. Finally, the reaction was detected with Classico and Crescendo Immobilon Western HRP Substrate kits (Millipore, Ref: WBLUC0100 and WBLUR0100). Film images were obtained using Ortho CP-G Plus (Agfa, Ref: EASUF) and Amersham Hyperfilm ECL (GE Healthcare, Ref: 28906837) films developed in an Amersham Hypercassette (GE Healthcare, Ref: RPN11643) and a Curix60 film developer (Agfa, Ref: 9462-1064095). Two biological replicates were performed. All used antibodies are listed in the tables below.

Flow cytometry was performed as previously described.^13^ Briefly, 500000 cells were resuspended in 200 µL PBS with BSA 0.5% (FC-Solution). 0.5 µL of FcR Blocking Reagent (Miltenyi Biotech, Ref: 130-059-901) was added and cells were incubated 10 minutes in ice. Then, 0.5 µL anti-ITGAX antibody conjugated to APC (see reference in table below) was added and cells were incubated 30 minutes in ice. Cells were washed once with 1 mL of FC-Solution, centrifuged 1000 g for 5 minutes and resuspended in 400 µL of FC-Solution. Finally, flow cytometry experiment was performed in a BD FACSCanto II (Becton Dickinson, Ref: 338962). Three biological replicates were performed. Antibodies are listed in the table below.

**5-aza-2’-deoxycytidine treatment of BLaER1 cells and qRT-PCR of validated genes**

BLaER1 cells were treated with 5-aza-2’-deoxycytidine (5-Aza) as previously described.^14^ Briefly, 2 x 10^6^ BLaER1 cells were cultured in 10 mL RPMI-1640 GlutaMAX, 10% FBS, 1X Penicillin/Streptomycin and two different concentrations of 5-Aza (0 µM and 1 µM). Cells were plated in 25 cm^2^ flasks in triplicate for each 5-Aza condition, incubated at 5% CO_2_, 37°C and harvested after 3 days of culture. Total RNA from frozen pellets was extracted, retrotranscribed and qRT-PCR was performed as described in the previous section “Validation of the Gene Expression changes” (Supplementary Methods).

**Chromosome capture with unique molecular identifiers (UMI-4C).**

UMI-4C was performed on ~4M cells before and after the transdifferentiation protocol. Cells were fixed with Formaldehyde 1%. Then, nuclei were digested with Csp6I and processed as previously described.^15^ Each library was obtained by nested PCRs and its molecular complexity was ensured by pooling 6 independent PCRs using the following primers: Downstream primer: GTTGTCCTTGGGTTTAGCTGC; Upstream primer: AGAAAGAGGAAGTCCTGGCAAT. Libraries were sequenced to a depth of >1M, 75bp long paired-end reads using either NextSeq or HiSeq 2500 platforms. UMI-4C sequencing reads were analyzed using in-house scripts compiled following the umi4cPackage.^15^ A Chi-squared test comparing UMIs chromatin contacts in a 4Kb windows centered on the transcriptional start site of the up-regulated genes annotated in the locus, was computed to identify differential chromatin contacts in cells that underwent, or not, the differentiation protocol.

**Transcription Factor Binding Site (TFBS) search in candidate CpG positions.**

In order to investigate the possibility that CpG methylation in our candidate positions could affect Transcription Factor (TF) binding, we performed an exhaustive search for putative TFBSs in the location of our candidate CpGs. We collect the -19/+20 bp sequences from the genomic positions of each of our 251 candidate CpGs (GCA_000001405.1, hg19 genome assembly) using bedtools (v2.28.0).^16^ Then, we use TFBSTools R package^17^ to search for motifs from JASPAR database^18^ in our collection of sequences. This tool performs the alignment of each of the input sequences (as well as their complementary reverse) with the position weight matrix (PWM) of each of the TFs in the selected database. Each alignment is scored with a percentage value representing the quantile between the minimal and the maximal possible value from the PWM. If present, we selected up to 5 alignments with a score over 90% as a potential TFBSs in our sequences.

In addition, a more specific search for CTCF binding sites was also conducting using CTCFBSDB2.0 prediction tool available at <http://insulatordb.uthsc.edu/storm_new.php>.^19^ This tool uses the STORM program^20^ and six selected PWM for CTCF binding sites to report the single best hit in the sequence. The PWM score corresponds to the log-odds of the observed sequence being generated by the motif versus being generated by the background. We selected sequences with a PWM score > 3.0, as suggested in the tool’s documentation, thus finding a total of 33 CpG sites with a putative CTCF binding site.

**Gene Ontology (GO) of the 80 Transcription factor with putative binding sites at CpG regions**

The set of 80 TFs with putative binding sites located at our 41 candidate promoter CpGs that correlated methylation ~ expression were used to compute overlaps with MSigDB (Molecular Signatures Database) GO Biological Process gene set collection, using online tools available at GSEA web site (<http://software.broadinstitute.org/>[gsea/msigdb/annotate.jsp](http://software.broadinstitute.org/gsea/msigdb/annotate.jsp)).^21,22^ This tool applies a hypergeometric test to find biological processes statistically over-represented in our gene set. Significance threshold was established at FDR < 0.05.

**References**

1. Rapino F, Robles EF, Richter-Larrea JA, Kallin EM, Martinez-Climent JA, Graf T. C/EBPα Induces Highly Efficient Macrophage Transdifferentiation of B Lymphoma and Leukemia Cell Lines and Impairs Their Tumorigenicity. Cell Rep. 2013;3(4):1153–1163.
2. Jin X, Kruth HS. Culture of Macrophage Colony-stimulating Factor Differentiated Human Monocyte-derived Macrophages. J Vis Exp. 2016 Jun 30;(112).
3. Rodríguez-Ubreva J, Català-Moll F, Obermajer N, Álvarez-Errico D, Ramirez RN, Company C, et al. Prostaglandin E2 Leads to the Acquisition of DNMT3A-Dependent Tolerogenic Functions in Human Myeloid-Derived Suppressor Cells. Cell Rep. 2017 Oct 3;21(1):154-167.
4. Aljanabi SM, Martinez I. Universal and rapid salt-extraction of high quality genomic DNA for PCR-based techniques. Nucleic Acids Res. 1997 Nov 15;25(22):4692-3.
5. Moran S, Arribas C, Esteller M. Validation of a DNA methylation microarray for 850,000 CpG sites of the human genome enriched in enhancer sequences. Epigenomics. 2016;8(3):389–399.
6. R Core Team. R: A language and environment for statistical computing. R Foundation for Statistical Computing, Vienna, Austria. https://www.R-project.org/. Accessed 17 June 2019.
7. Aryee MJ, Jaffe AE, Corrada-Bravo H, Ladd-Acosta C, Feinberg AP, Hansen KD, et al. Minfi: A flexible and comprehensive Bioconductor package for the analysis of Infinium DNA Methylation microarrays. Bioinformatics. 2014;30(10):1363-1369.
8. Fortin JP, Triche TJ, Hansen KD. Preprocessing, normalization and integration of the Illumina HumanMethylationEPIC array with minfi. Bioinformatics. 2017;33(4):558-560
9. Pidsley R, Zotenko E, Peters TJ, Lawrence MG, Risbridger GP, Molloy P, et al. Critical evaluation of the Illumina MethylationEPIC BeadChip microarray for whole-genome DNA. Genome Biol. 2016;17(1):208.
10. Du P, Zhang X, Huang CC, Jafari N, Kibbe WA, Hou L, et al. Comparison of Beta-value and M-value methods for quantifying methylation levels by microarray analysis. BMC Bioinformatics. 2010 Nov 30;11:587.
11. Chain B. agilp: Agilent expression array processing package. R package version 3.14.0. [http://www.bioconductor.org/packages //2.12/bioc/html/agilp.html. Accessed 20 June 2019](http://www.bioconductor.org/packages%20//2.12/bioc/html/agilp.html.%20Accessed%2020%20June%202019).
12. Szczesna K, de la Caridad O, Petazzi P, Soler M, Roa L, Saez MA, et al. Improvement of the Rett syndrome phenotype in a MeCP2 mouse model upon treatment with levodopa and a dopa-decarboxylase inhibitor. Neuropsychopharmacology. 2014 Nov;39(12):2846-56
13. Azagra A, Román-González L, Collazo O, Rodríguez-Ubreva J, de Yébenes VG, Barneda-Zahonero B, et al. In vivo conditional deletion of HDAC7 reveals its requirement to establish proper B lymphocyte identity and development. J Exp Med. 2016 Nov 14;213(12):2591-2601.
14. Janin M, Ortiz-Barahona V, de Moura MC, Martínez-Cardús A, Llinàs-Arias P, Soler M, et al. Epigenetic loss of RNA-methyltransferase NSUN5 in glioma targets ribosomes to drive a stress adaptive translational program. Acta Neuropathol. 2019 Aug 19.
15. Schwartzman O, Mukamel Z, Oded-Elkayam N, Olivares-Chauvet P, Lubling Y, Landan G, et al. UMI-4C for quantitative and targeted chromosomal contact profiling. Nat Methods. 2016;13(8):685-91.
16. Quinlan AR, Hall IM. BEDTools: a flexible suite of utilities for comparing genomic features. Bioinformatics. 2010 Mar 15;26(6):841-2.
17. Tan G, Lenhard B. TFBSTools: an R/bioconductor package for transcription factor binding site analysis. Bioinformatics. 2016 May 15;32(10):1555-6.
18. Khan A, Fornes O, Stigliani A, Gheorghe M, Castro-Mondragon JA, van der Lee R, et al. JASPAR 2018: update of the open-access database of transcription factor binding profiles and its web framework. Nucleic Acids Res. 2018 Jan 4;46(D1):D1284.
19. Ziebarth JD, Bhattacharya A, Cui Y. CTCFBSDB 2.0: a database for CTCF-binding sites and genome organization. Nucleic Acids Res. 2013 Jan;41(Database issue):D188-94.
20. Schones DE, Smith AD, Zhang MQ. Statistical significance of cis-regulatory modules. BMC Bioinformatics. 2007 Jan 22;8:19.
21. Subramanian A, Tamayo P, Mootha VK, Mukherjee S, Ebert BL, Gillette MA, et al. Gene set enrichment analysis: a knowledge-based approach for interpreting genome-wide expression profiles. Proc Natl Acad Sci U S A. 2005 Oct 25;102(43):15545-50.
22. Mootha VK, Lindgren CM, Eriksson KF, Subramanian A, Sihag S, Lehar J, et al. PGC-1alpha-responsive genes involved in oxidative phosphorylation are coordinately downregulated in human diabetes. Nat Genet. 2003 Jul;34(3):267-73.

**Table. Primers and antibodies used for quantitative real-time PCR (qRT-PCR), bisulfite sequencing PCR (BSP), Pyrosequencing, Western Blot and Flow Citometry analysis.**

| **Primers qRT-PCR** | **Forward (5' to 3')** | | | **Reverse (5' to 3')** | | | |
| --- | --- | --- | --- | --- | --- | --- | --- |
| IL1RN | TGAGGACCAGCCATTG | | | AGACCATTCTGGAGGCAG | | | |
| ITGAX | CGTTCGACACATCCGTGTA | | | TTTGCCTCCTCCATCATTTC | | | |
| RHOG | CTCTCACTTCCTTCTCGAGCC | | | GTTGCTGTAGTGGAGGCAGT | | | |
| CCR1 | GCATGAACTCTCTGCTGGGT | | | CCCCAGGCCACCATTACATT | | | |
| CXCL8 | TGGACCCCAAGGAAAACTGG | | | TGGCATCTTCACTGATTCTTGGA | | | |
| CHML | GCAGGAGGTTTAATATTGATTTG | | | AAGACATCTGCTCTGGAACA | | | |
| DBF4 | TACCTTCTGTCACCATATCTGAA | | | TCGACCCAAGGTTTGTG | | | |
| PGK | CTGGGCAAGGATGTTCTGTT | | | CACATGAAAGCGGAGGTTCT | | | |
|  |  | | |  | | | |
| **Primers BSP** | **Forward (5' to 3')** | | | **Reverse (5' to 3')** | | | |
| ITGAX promoter | TTAAGGGTGAGTTTGGGA | | | CTACCCTAATCCTAATCATAACTAAAA | | | |
| IL1RN promoter | AGGGGAGGGAATTAGTTATAAT | | | AAAACCTCTACAAATTTCCATTC | | | |
| RHOG related distant region | GGGGGTTTGATGAGATAAGG | | | AAACCACCACACCTAACCTAAA | | | |
| CCR1 related distant region | TTTGTAAGTTAGGAAGGGGGATA | | | CATCAAAACAAAACTCCATCTCA | | | |
| CXCL8 related distant region | TAGAAGTAAATGAAGTTTGGGTTGG | | | CCAAATATCTTCTCCCCACCA | | | |
| CHML related distant region | TGAGGGTAATTTTGGGGATTT | | | AAAATCACACTCCCTCAACACA | | | |
| DBF4 related distant region | TTTTAAAATTTGTTGAAAGGGATG | | | TTCAAAACAATATATTATCCCAAAACC | | | |
|  |  | | |  | | | |
| **Primers PCR Pyrosequencing** | **Forward (5’ to 3’)** | **Reverse (5’ to 3’)** | | | | **Sequencing**  **(5’ to 3’)** | |
| ITGAX promoter | AGGGTATTAAGTTAAGTTATTTGATGAGA | Biotin(5’)-ACCCTAATCCTAATCATAACTAAAAAATC | | | | AGTGGGGTTGAAAGTGATAAT | |
| IL1RN promoter | Biotin(5’)-AGTGGGGTTGAAAGTGATAAT | AAAACCTCTACAAATTTCCATTCTA | | | | CACTCACCCAAACTAA | |
| RHOG related distant region | TGTAAATGGTGGTTTTTATTAAAAGTGAAG | Biotin(5’)-CACCTCAACCTCCCAAAAT | | | | AGGTATTTATTTTATGTAGAA | |
| CCR1 related distant region | Biotin(5’)-AGTTAGGAAGGGGGATATTATTAG | TACCAAAACTTCCTCTTTCTTCAC | | | | CCTCTTTCTTCACCTAC | |
| CXCL8 related distant region | Biotin(5’)-GGTTGGGTTTAAATTTATTGTGGTAAAG | ACAACTTCTCCCCATAAAACACATCATT | | | | AAAACACATCATTAAAAAATAACTA | |
| CHML related distant region | GGGGATTGTTTATTTAGATTTAGTAGT | Biotin(5’)-ACTCCCTCAACACAATACTT | | | | AGAAAAGAGGAATAGGTTAT | |
| DBF4 related distant region | GTTTTAAAATTTGTTGAAAGGGATGTT | Biotin(5’)-ATTCAAAACAATATATTATCCCAAAACC | | | | TGTTAATTGTAGTGGTTAATTTT | |
|  | | | | | | | |
| **Primers UMI-4C** | **Upstream Primer** | | **Downstream Primer** | | | | |
| CCR1 | GTTGTCCTTGGGTTTAGCTGC | | AGAAAGAGGAAGTCCTGGCAAT | | | | |
|  |  | | |  | | | |
| **Antibodies** | **Company** | | **Reference** | | **Source** | | **Dilution** |
| IL1RN | R&D Systems | | AF-280-NA | | Goat | | 1:400 |
| ITGAX-APC | BD Pharmingen | | 559877 | | Mouse | | 1:400 |
| DNMT1 | Boster | | CI1105 | | Rabbit | | 1:500 |
| DNMT3A | Abcam | | ab2850 | | Rabbit | | 1:500 |
| DNMT3B | Sigma-Aldrich | | HPA001595 | | Rabbit | | 1:500 |
| Lamin B1 | Abcam | | ab16048 | | Rabbit | | 1:5000 |
| Anti-Rabbit | Sigma-Aldrich | | A0545 | | Goat | | 1:10000 |
| Anti-Goat | Dako | | P0449 | | Rabbit | | 1:5000 |

**PCR conditions for qRT-PCR, BSP and Pyrosequencing**

| **qRT-PCR conditions** | | | | | | | | | | | |
| --- | --- | --- | --- | --- | --- | --- | --- | --- | --- | --- | --- |
| **Hold Stage** | | | **PCR Stage (40 cycles)** | | | | **Melting Curve Stage** | | | | |
| 50 °C  2 min | 95 °C  10 min | | 95 °C  15 sec | | 60 °C  1 min | | 95 °C  15 sec | | 60 °C  1 min | | 95 °C  15 sec |
|  | | | | | | | | | | | |
| **BSP conditions (for 100 ng DNA)** | | | | | | | | | | | |
| **Hold Stage** | | **PCR Stage (40 cycles)** | | | | | | **Resting Stage** | | | |
| 98 °C  1 min | | 98 °C  10 sec | | 57 °C  45 sec | | 72 °C  1 min | | 72 °C  7 min | | 4 °C  ∞ | |
|  | | | | | | | | | | | |
| **Pyrosequencing PCR conditions (for 10 ng DNA)** | | | | | | | | | | | |
| **Hold Stage** | | **PCR Stage (48 cycles)** | | | | | | **Resting Stage** | | | |
| 96 °C  10 min | | 96 °C  30 sec | | 58 °C  30 sec | | 72 °C  30 sec | | 72 °C  10 min | | 15 °C  ∞ | |

**Genomic regions of the demethylated CpGs under study. Genomic nucleotidic position correspond to the hg19 human genome version.**

| **CpG Regions** | **CpG code** | **Genomic Position** |
| --- | --- | --- |
| IL1RN promoter | cg02543462 | chr2:113885116 |
| ITGAX promoter | cg20851120 | chr16:31366406 |
|  | cg04742550 | chr16:31366429 |
| RHOG related distant region | cg21880051 | chr11:9661734 |
| CCR1 related distant region | cg21655255 | chr3:46136952 |
| CXCL8 related distant region | cg05146536 | chr4:77140678 |
| CHML related distant region | cg00250658 | chr1:223349112 |
| DBF4 related distant region | cg06297012 | chr7:90929852 |
|  | | |
| **BSP Regions** | **From (5’ to 3’)** | **To (5’ to 3’)** |
| IL1RN promoter | chr2:113884986 | chr2:113885222 |
| ITGAX promoter | chr16:31366289 | chr16:31366594 |
| RHOG related distant region | chr11:9661553 | chr11:9661894 |
| CCR1 related distant region | chr3:46136807 | chr3:46137151 |
| CXCL8 related distant region | chr4:77140560 | chr4:77140859 |
| CHML related distant region | chr1:223348914 | chr1:223349150 |
| DBF4 related distant region | chr7:90929660 | chr7:90929889 |
|  | | |
| **Pyrosequencing Regions** | **From (5’ to 3’)** | **To (5’ to 3’)** |
| IL1RN promoter | chr2:113885083 | chr2:113885112 |
| ITGAX promoter | chr16:31366405 | chr16:31366459 |
| RHOG related distant region | chr11:9661726 | chr11:9661761 |
| CCR1 related distant region | chr3:46136912 | chr3:46136958 |
| CXCL8 related distant region | chr4:77140639 | chr4:77140687 |
| CHML related distant region | chr1:223349092 | chr1:223349138 |
| DBF4 related distant region | chr7:9092983 | chr7:90929881 |

**Links to DNA Methylation microarray and UMI-4C data repositories**

DNA Methylation data (GEO-GSE132845)

- <https://www.ncbi.nlm.nih.gov/geo/query/acc.cgi?acc=GSE132845>

UMI-4C data (SRA-PRJNA548887):

- <https://dataview.ncbi.nlm.nih.gov/object/PRJNA548887?reviewer=6r4e14p2u96d32rgr3fqtpda8c>
