# Supplementary material for: B-cell leukemia transdifferentiation to macrophage involves reconfiguration of DNA methylation for long-range regulation
Source: Leukemia. 2019 Nov 12;34(4):1158–62. doi: 10.1038/s41375-019-0643-1 (PMC7214273; doi:10.1038/s41375-019-0643-1)
Supplement: Supplementary file 2 — Supplementary Figure 1 [file 41375_2019_643_MOESM2_ESM.pptx]

## Slide 1
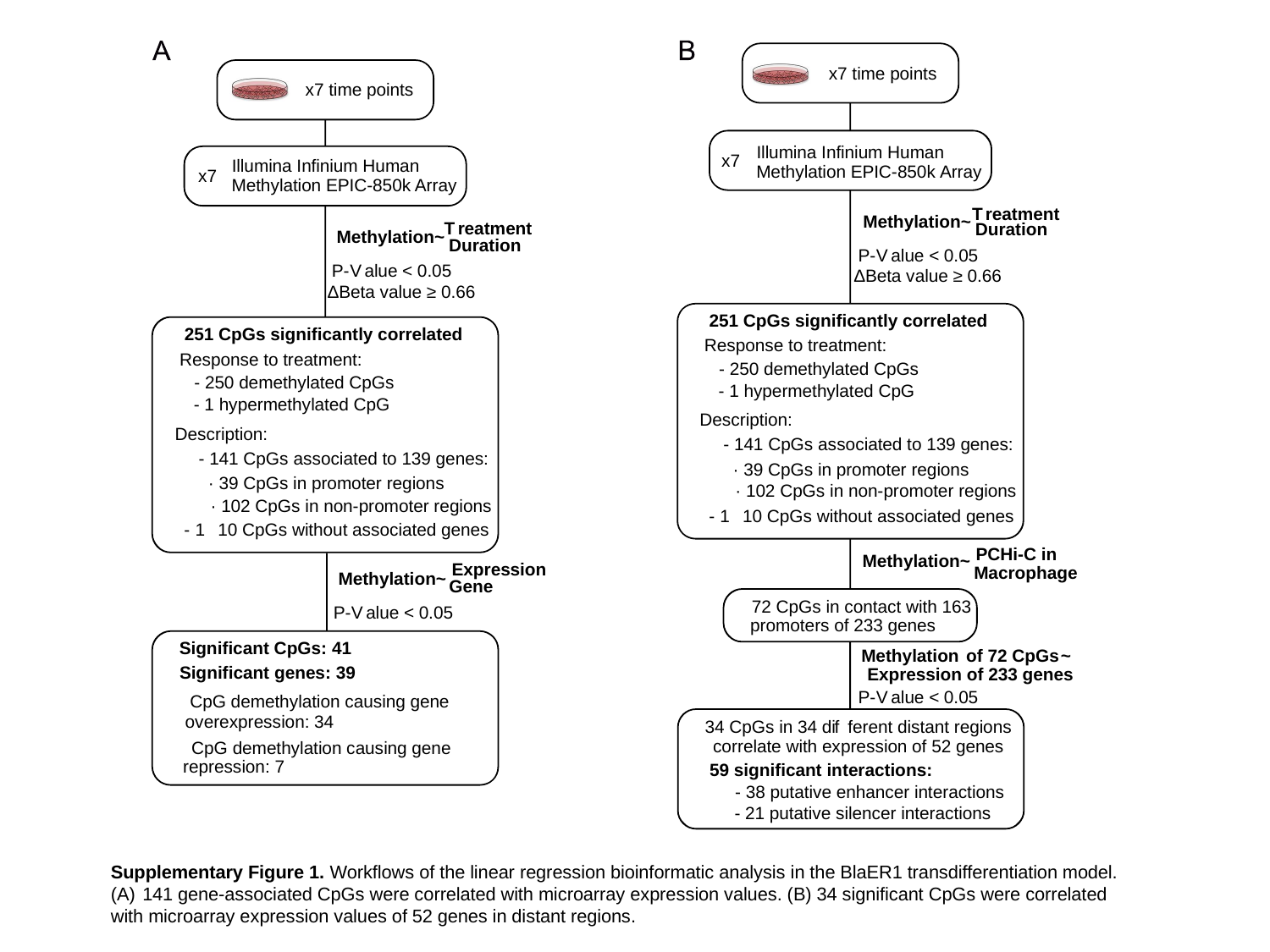

x7 time points
x7 time points
Illumina Infinium Human
x7
Illumina Infinium Human
Methylation EPIC-850k
Array
x7
Methylation EPIC-850k
Array
T
reatment
Methylation~
T
reatment
Duration
Methylation~
Duration
P-
V
alue < 0.05
P-
V
alue < 0.05
ΔBeta value ≥ 0.66
ΔBeta value ≥ 0.66
251 CpGs significantly correlated
251 CpGs significantly correlated
Response to treatment:
Response to treatment:
- 250 demethylated CpGs
- 250 demethylated CpGs
- 1 hypermethylated CpG
- 1 hypermethylated CpG
Description:
Description:
- 141 CpGs associated to 139 genes:
- 141 CpGs associated to 139 genes:
· 39 CpGs in promoter regions
· 39 CpGs in promoter regions
· 102 CpGs in non-promoter regions
· 102 CpGs in non-promoter regions
-
1
10 CpGs without associated genes
-
1
10 CpGs without associated genes
PCHi-C in
Methylation~
Expression
Macrophage
Methylation~
Gene
72 CpGs in contact with 163
P-
V
alue < 0.05
promoters of 233 genes
Significant CpGs: 41
~
Methylation
of 72 CpGs
Significant genes: 39
Expression of 233 genes
P-
V
alue < 0.05
CpG demethylation causing gene
overexpression: 34
34 CpGs in 34 di
f
ferent distant regions
correlate with expression of 52 genes
CpG demethylation causing gene
repression: 7
59 significant interactions:
- 38 putative enhancer interactions
- 21 putative silencer interactions
Supplementary Figure 1. Workflows of the linear regression bioinformatic analysis in the BlaER1 transdifferentiation model.
141 gene-associated CpGs were correlated with microarray expression values. (B) 34 significant CpGs were correlated
with microarray expression values of 52 genes in distant regions.
