# Supplementary material for: B-cell leukemia transdifferentiation to macrophage involves reconfiguration of DNA methylation for long-range regulation
Source: Leukemia. 2019 Nov 12;34(4):1158–62. doi: 10.1038/s41375-019-0643-1 (PMC7214273; doi:10.1038/s41375-019-0643-1)
Supplement: Supplementary file 3 — Supplementary Figure 2 [file 41375_2019_643_MOESM3_ESM.pptx]

## Slide 1
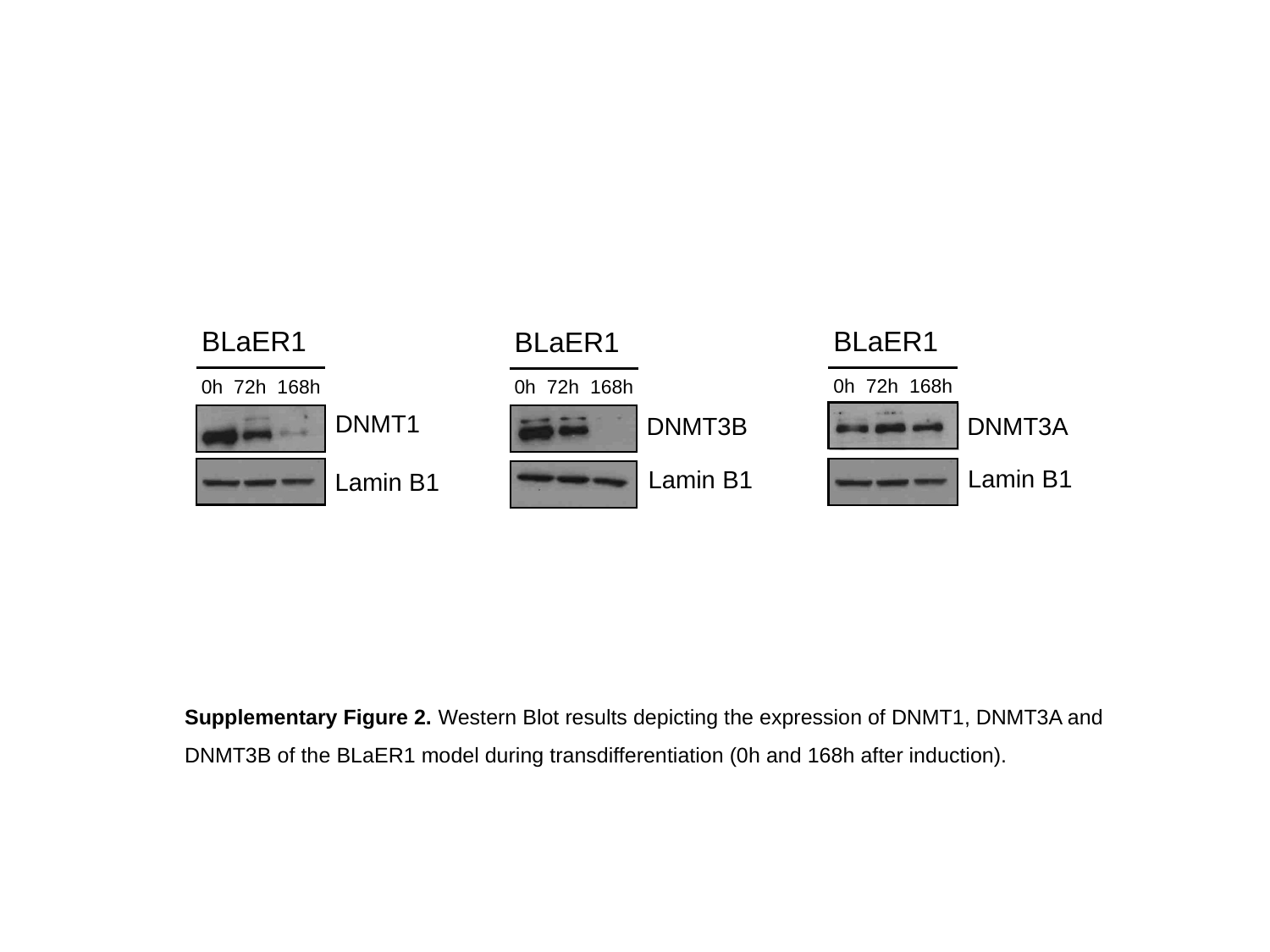

BLaER1
BLaER1
BLaER1
 0h 72h 168h
 0h 72h 168h
 0h 72h 168h
DNMT1
DNMT3B
DNMT3A
Lamin B1
Lamin B1
Lamin B1
Supplementary Figure 2. Western Blot results depicting the expression of DNMT1, DNMT3A and
DNMT3B of the BLaER1 model during transdifferentiation (0h and 168h after induction).
