# Supplementary material for: B-cell leukemia transdifferentiation to macrophage involves reconfiguration of DNA methylation for long-range regulation
Source: Leukemia. 2019 Nov 12;34(4):1158–62. doi: 10.1038/s41375-019-0643-1 (PMC7214273; doi:10.1038/s41375-019-0643-1)
Supplement: Supplementary file 4 — Supplementary Figure 3 [file 41375_2019_643_MOESM4_ESM.pptx]

## Slide 1
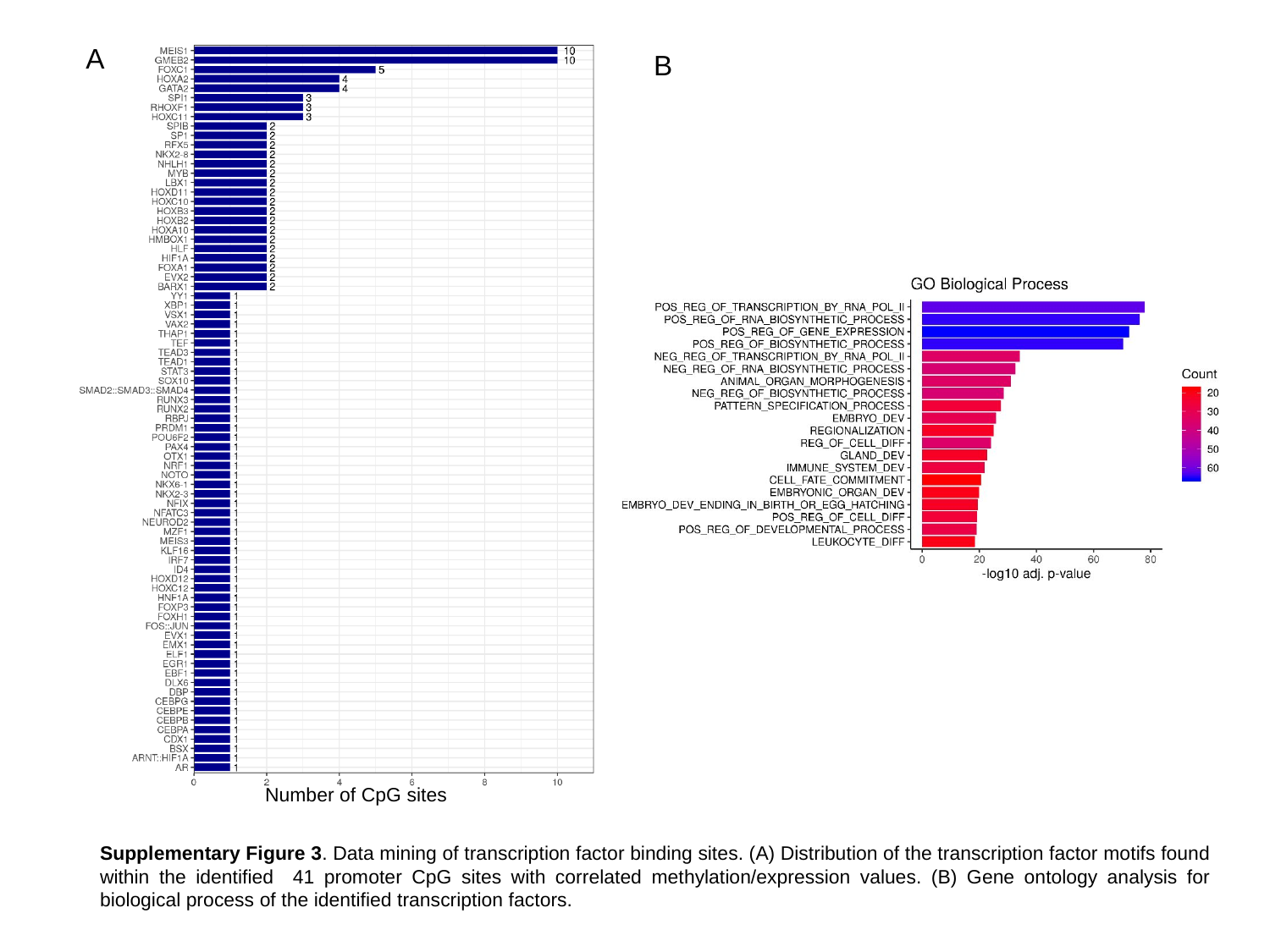

A
B
Number of CpG sites
Supplementary Figure 3. Data mining of transcription factor binding sites. (A) Distribution of the transcription factor motifs found within the identified 41 promoter CpG sites with correlated methylation/expression values. (B) Gene ontology analysis for biological process of the identified transcription factors.
