# Supplementary material for: B-cell leukemia transdifferentiation to macrophage involves reconfiguration of DNA methylation for long-range regulation
Source: Leukemia. 2019 Nov 12;34(4):1158–62. doi: 10.1038/s41375-019-0643-1 (PMC7214273; doi:10.1038/s41375-019-0643-1)
Supplement: Supplementary file 5 — Supplementary Figure 4 [file 41375_2019_643_MOESM5_ESM.pptx]

## Slide 1
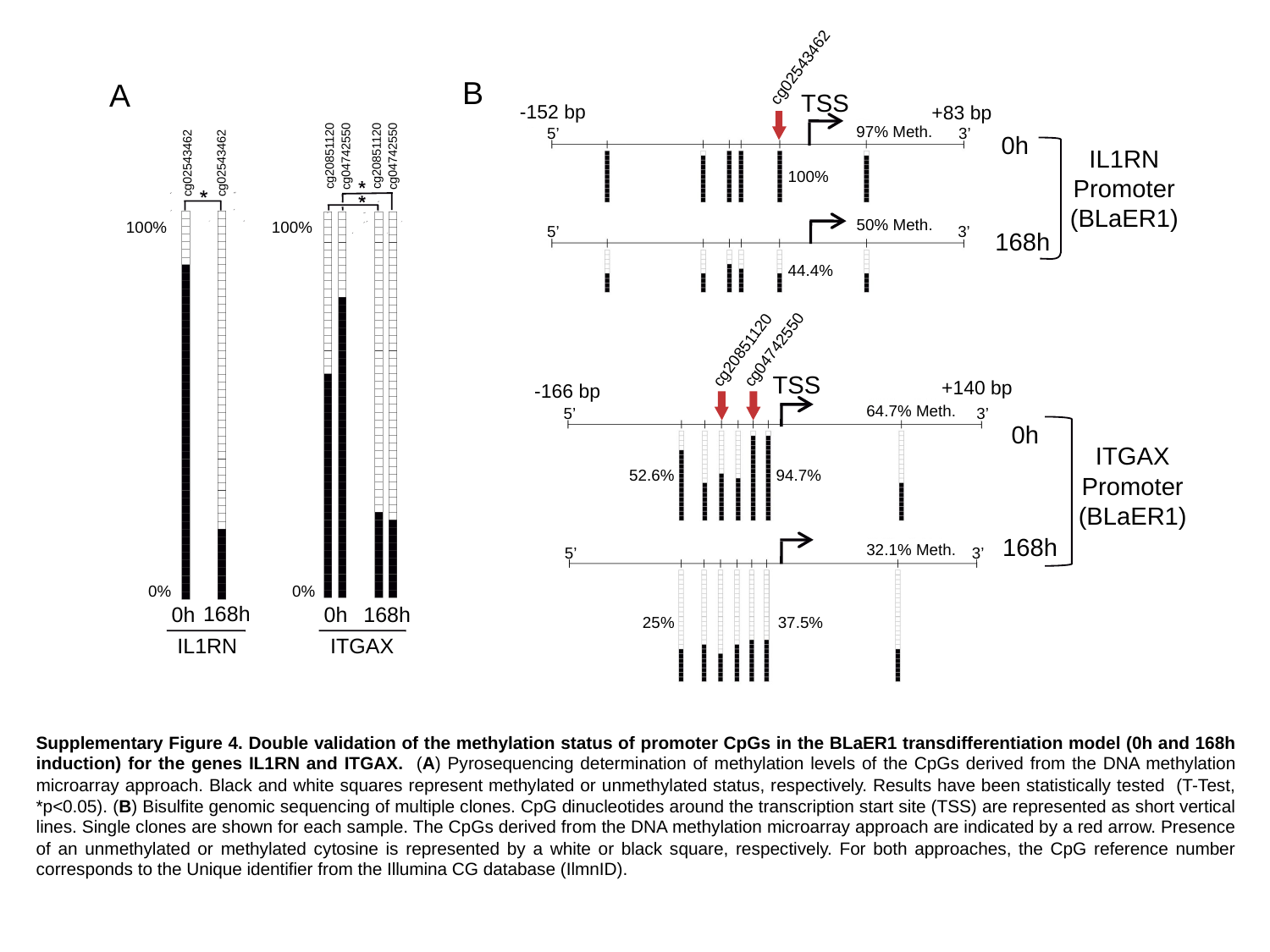

cg02543462
B
A
TSS
-152 bp
+83 bp
97% Meth.
3’
5’
0h
IL1RN
Promoter
(BLaER1)
cg20851120
cg04742550
cg20851120
cg04742550
cg02543462
cg02543462
100%
50% Meth.
100%
100%
3’
5’
168h
44.4%
cg20851120
cg04742550
TSS
+140 bp
-166 bp
64.7% Meth.
5’
3’
0h
ITGAX
Promoter
(BLaER1)
52.6%
94.7%
168h
32.1% Meth.
5’
3’
0%
0%
168h
0h
0h
168h
25%
37.5%
IL1RN
ITGAX
Supplementary Figure 4. Double validation of the methylation status of promoter CpGs in the BLaER1 transdifferentiation model (0h and 168h induction) for the genes IL1RN and ITGAX. (A) Pyrosequencing determination of methylation levels of the CpGs derived from the DNA methylation microarray approach. Black and white squares represent methylated or unmethylated status, respectively. Results have been statistically tested (T-Test, *p<0.05). (B) Bisulfite genomic sequencing of multiple clones. CpG dinucleotides around the transcription start site (TSS) are represented as short vertical lines. Single clones are shown for each sample. The CpGs derived from the DNA methylation microarray approach are indicated by a red arrow. Presence of an unmethylated or methylated cytosine is represented by a white or black square, respectively. For both approaches, the CpG reference number corresponds to the Unique identifier from the Illumina CG database (IlmnID).
