# Supplementary material for: B-cell leukemia transdifferentiation to macrophage involves reconfiguration of DNA methylation for long-range regulation
Source: Leukemia. 2019 Nov 12;34(4):1158–62. doi: 10.1038/s41375-019-0643-1 (PMC7214273; doi:10.1038/s41375-019-0643-1)
Supplement: Supplementary file 6 — Supplementary Figure 5 [file 41375_2019_643_MOESM6_ESM.pptx]

## Slide 1
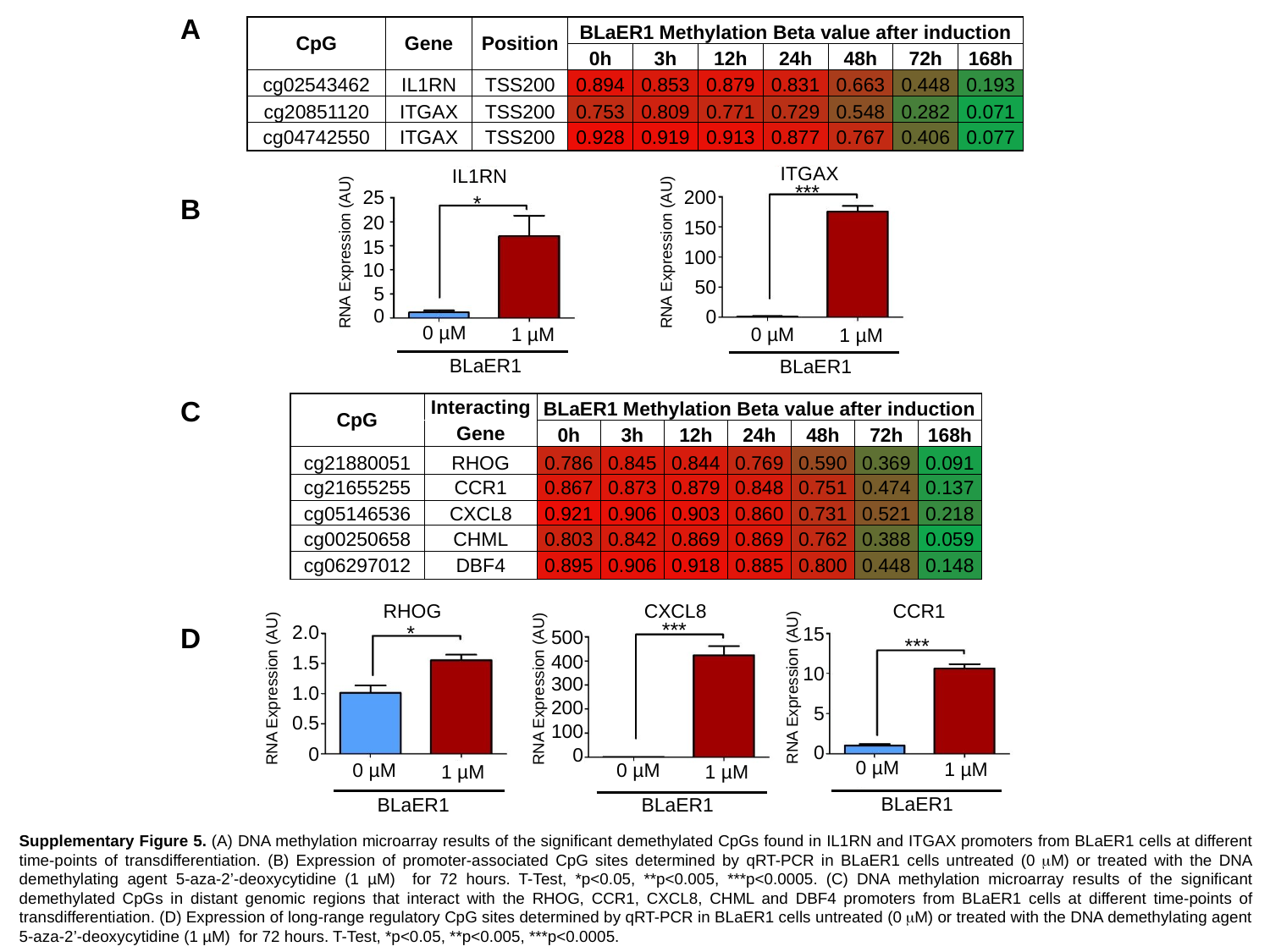

A
| CpG | Gene | Position | BLaER1 Methylation Beta value after induction | | | | | | |
| --- | --- | --- | --- | --- | --- | --- | --- | --- | --- |
| | | | 0h | 3h | 12h | 24h | 48h | 72h | 168h |
| cg02543462 | IL1RN | TSS200 | 0.894 | 0.853 | 0.879 | 0.831 | 0.663 | 0.448 | 0.193 |
| cg20851120 | ITGAX | TSS200 | 0.753 | 0.809 | 0.771 | 0.729 | 0.548 | 0.282 | 0.071 |
| cg04742550 | ITGAX | TSS200 | 0.928 | 0.919 | 0.913 | 0.877 | 0.767 | 0.406 | 0.077 |
ITGAX
IL1RN
***
25
200
*
B
20
150
15
RNA Expression (AU)
RNA Expression (AU)
100
10
50
5
0
0
0 µM
1 µM
0 µM
1 µM
BLaER1
BLaER1
C
| CpG | Interacting | BLaER1 Methylation Beta value after induction | | | | | | |
| --- | --- | --- | --- | --- | --- | --- | --- | --- |
| | Gene | 0h | 3h | 12h | 24h | 48h | 72h | 168h |
| cg21880051 | RHOG | 0.786 | 0.845 | 0.844 | 0.769 | 0.590 | 0.369 | 0.091 |
| cg21655255 | CCR1 | 0.867 | 0.873 | 0.879 | 0.848 | 0.751 | 0.474 | 0.137 |
| cg05146536 | CXCL8 | 0.921 | 0.906 | 0.903 | 0.860 | 0.731 | 0.521 | 0.218 |
| cg00250658 | CHML | 0.803 | 0.842 | 0.869 | 0.869 | 0.762 | 0.388 | 0.059 |
| cg06297012 | DBF4 | 0.895 | 0.906 | 0.918 | 0.885 | 0.800 | 0.448 | 0.148 |
RHOG
CXCL8
CCR1
***
2.0
*
D
15
500
***
400
1.5
10
300
RNA Expression (AU)
RNA Expression (AU)
RNA Expression (AU)
1.0
200
5
0.5
100
0
0
0
0 µM
1 µM
0 µM
0 µM
1 µM
1 µM
BLaER1
BLaER1
BLaER1
Supplementary Figure 5. (A) DNA methylation microarray results of the significant demethylated CpGs found in IL1RN and ITGAX promoters from BLaER1 cells at different time-points of transdifferentiation. (B) Expression of promoter-associated CpG sites determined by qRT-PCR in BLaER1 cells untreated (0 mM) or treated with the DNA demethylating agent 5-aza-2’-deoxycytidine (1 µM) for 72 hours. T-Test, *p<0.05, **p<0.005, ***p<0.0005. (C) DNA methylation microarray results of the significant demethylated CpGs in distant genomic regions that interact with the RHOG, CCR1, CXCL8, CHML and DBF4 promoters from BLaER1 cells at different time-points of transdifferentiation. (D) Expression of long-range regulatory CpG sites determined by qRT-PCR in BLaER1 cells untreated (0 mM) or treated with the DNA demethylating agent 5-aza-2’-deoxycytidine (1 µM) for 72 hours. T-Test, *p<0.05, **p<0.005, ***p<0.0005.
