# Supplementary material for: B-cell leukemia transdifferentiation to macrophage involves reconfiguration of DNA methylation for long-range regulation
Source: Leukemia. 2019 Nov 12;34(4):1158–62. doi: 10.1038/s41375-019-0643-1 (PMC7214273; doi:10.1038/s41375-019-0643-1)
Supplement: Supplementary file 7 — Supplementary Figure 6 [file 41375_2019_643_MOESM7_ESM.pptx]

## Slide 1
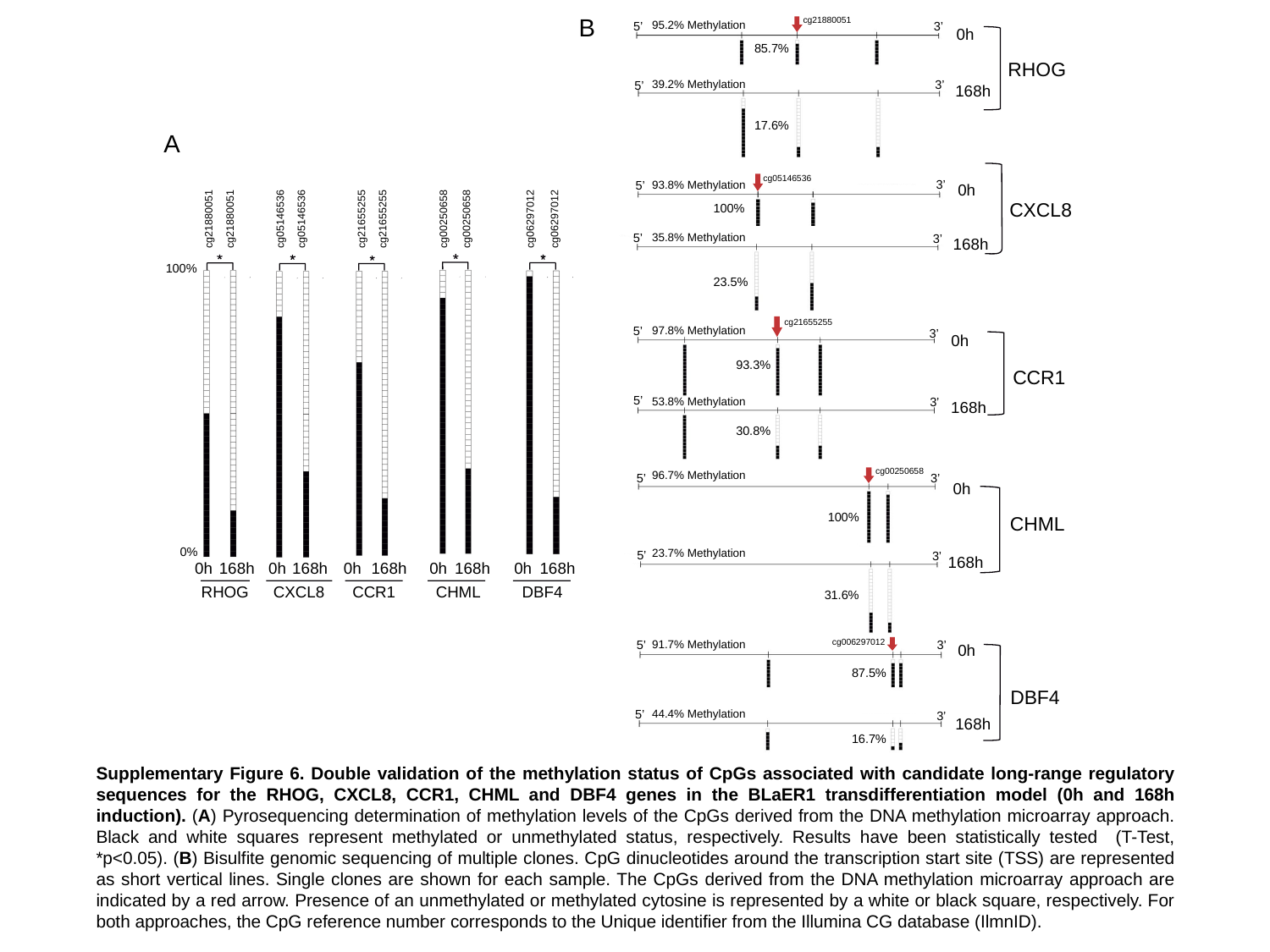

B
95.2% Methylation
5’
3’
cg21880051
0h
85.7%
RHOG
39.2% Methylation
3’
5’
168h
17.6%
A
3’
93.8% Methylation
5’
cg05146536
0h
CXCL8
100%
cg21880051
cg21880051
cg05146536
cg05146536
cg21655255
cg21655255
cg00250658
cg00250658
cg06297012
cg06297012
35.8% Methylation
5’
3’
168h
100%
23.5%
cg21655255
5’
97.8% Methylation
3’
0h
93.3%
CCR1
5’
3’
53.8% Methylation
168h
30.8%
96.7% Methylation
3’
5’
cg00250658
0h
100%
CHML
0%
23.7% Methylation
5’
3’
168h
0h
168h
0h
168h
0h
168h
0h
168h
0h
168h
RHOG
CXCL8
CCR1
CHML
DBF4
31.6%
3’
91.7% Methylation
5’
0h
cg006297012
87.5%
DBF4
44.4% Methylation
5’
3’
168h
16.7%
Supplementary Figure 6. Double validation of the methylation status of CpGs associated with candidate long-range regulatory sequences for the RHOG, CXCL8, CCR1, CHML and DBF4 genes in the BLaER1 transdifferentiation model (0h and 168h induction). (A) Pyrosequencing determination of methylation levels of the CpGs derived from the DNA methylation microarray approach. Black and white squares represent methylated or unmethylated status, respectively. Results have been statistically tested (T-Test, *p<0.05). (B) Bisulfite genomic sequencing of multiple clones. CpG dinucleotides around the transcription start site (TSS) are represented as short vertical lines. Single clones are shown for each sample. The CpGs derived from the DNA methylation microarray approach are indicated by a red arrow. Presence of an unmethylated or methylated cytosine is represented by a white or black square, respectively. For both approaches, the CpG reference number corresponds to the Unique identifier from the Illumina CG database (IlmnID).
